# Safety and efficacy of endobronchial ultrasound‐guided transbronchial needle aspiration (EBUS‐TBNA) for patients aged 80 years and older

**DOI:** 10.1111/1759-7714.14454

**Published:** 2022-05-06

**Authors:** Hideyuki Niwa, Masahide Oki, Yurika Ishii, Atsushi Torii, Arisa Yamada, Yuka Shinohara, Yoshihito Kogure, Hideo Saka

**Affiliations:** ^1^ National Hospital Organization Nagoya Medical Center, Department of Respiratory Medicine Nagoya Japan; ^2^ Department of Respiratory Medicine Nagoya University Nagoya Japan

**Keywords:** endobronchial ultrasonography, geriatric oncology, non‐small cell lung cancer, bronchoscopy, small cell lung cancer

## Abstract

**Background:**

The safety and efficacy of endobronchial ultrasound‐guided transbronchial needle aspiration (EBUS‐TBNA) in patients aged 70 years and older has been established. However, few studies have evaluated the safety, usefulness, and significance of EBUS‐TBNA in patients aged 80 years and older.

**Methods:**

We retrospectively investigated patients aged 80 years and older who underwent EBUS‐TBNA under local anesthesia. The study period was 10 years; from November 1, 2010 to October 31, 2020. The primary endpoint was the safety of EBUS‐TBNA, which was measured as the incidence of complications associated with the procedure. The secondary endpoints were the overall diagnostic rate, malignant disease diagnosis rate, and malignant disease treatment rate.

**Results:**

A total of 111 patients were enrolled in the study, and the median age was 82 years (range: 80–89 years). The incidence of complications (the primary endpoint) was 5% (5/111) and comprised oversedation in one case, chest pain in one case, hypoxemia in two cases, and arrhythmia in one case. Regarding the secondary endpoints, the diagnostic rate for all patients was 75% (83/111), and the diagnostic rate of malignant disease was 89% (75/84). Of the 75 patients with malignant disease diagnosed with EBUS‐TBNA, 61 (81%) received tumor‐specific therapy in accordance with their diagnoses.

**Conclusion:**

EBUS‐TBNA can be considered safe and effective even in patients aged 80 years and older.

## Graphical abstract

A total of 111 patients were enrolled in the study. The incidence of complications was 5%. The diagnosyic rate for all patients was 75%. The diagnostic rate of malignant disease was 89%. Of the 75 patients with malignant disease diagnosed with EBUS‐TBNA, 61 (81%) received tumor‐specific therapy in accordance with their diagnoses. This study suggests that EBUS‐TBNA could be performed without restrictions in people older than 80 years.

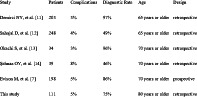

## INTRODUCTION

Lung cancer has a particularly poor prognosis and a high mortality rate among patients with malignant disease.^1^ When metastasis to mediastinal lymph nodes or hilar lymph nodes is identified, there are few indications for surgery, and drug therapy and radiation therapy are the mainstay of treatment. However, safety and efficacy of drug therapy have gradually been demonstrated for the treatment of elderly lung cancer patients.^2^, ^3^ Recently, the efficacy of tyrosine kinase inhibitors and immune checkpoint inhibitors that target driver gene mutations have been demonstrated,^4^, ^5^ and the prognosis of lung cancer is expected to improve as a result, even in the elderly. Therefore, there is a need for safer and more effective biopsy methods in the treatment of lung cancer in the elderly.

Endobronchial ultrasound‐guided transbronchial needle aspiration (EBUS‐TBNA) is a widely used technique for the biopsy of neoplastic lesions in the mediastinum, hilar lymph nodes, and lung fields owing to its high efficacy and safety.^6^ Studies of EBUS‐TBNA in elderly patients have been performed, and the safety and efficacy of the procedure has been shown in prospective studies in patients over 70 years of age.^7^ In contrast, the safety and significance of EBUS‐TBNA in patients older than 80 years of age have not been clarified. Additionally, one report found that flexible bronchoscopy in patients older than 80 years of age was associated with more complications than the rate in younger patients.^8^ If the safety of EBUS‐TBNA in patients older than 80 years of age is not guaranteed, this procedure cannot be performed in these patients and they may not receive appropriate treatment. Therefore, in this study, we investigated the safety and efficacy of EBUS‐TBNA by retrospectively evaluating patients over 80 years of age who underwent EBUS‐TBNA for diagnostic purposes.

## METHODS

### Patients

During the 10‐year period from November 1, 2010 to October 31, 2020, 1597 patients underwent EBUS‐TBNA at Nagoya Medical Center, Aichi, Japan. Among the 1597 patients, we selected patients older than 80 years of age. Patients who underwent simultaneous transesophageal biopsy by fine needle aspiration and those who underwent EBUS‐TBNA for staging purposes were excluded. Patient information was reviewed retrospectively from the medical records, and written informed consent was obtained from all patients before EBUS‐TBNA was performed. This study was conducted in accordance with the World Medical Association Declaration of Helsinki. In addition, this study was approved by the National Hospital Organization Nagoya Medical Center Institutional Review Board (approval date: May 14, 2021; identifier: 2020–103).

### Procedures

All patients underwent EBUS‐TBNA orally under local anesthesia. Methods of local anesthesia are described below. In our institution, midazoram 0.03–0.06 mg/kg and fentanyl 0.8–1 μg/kg are initially administered. Specifically, 10 mg midazolam and 100 μg fentanyl were diluted to 10 ml with saline and administered during the procedure. The dose was increased at the discretion of the bronchoscopist according to the patient's anesthetic depth (to maintain less than +1 on the RASS scale). SpO_2_ and heart rate were monitored in all patients and oxygen was administered to keep the SpO2 above 90% during EBUS‐TBNA.

The method of EBUS‐TBNA was in accordance with the British Thoracic Society guidelines,^9^ apart from the size of the needle, number of biopsies, and biopsy sites, which were left to the attending physician's discretion. Bronchoscopes used were the Olympus BF‐UC290F (Olympus), BF‐UC260‐OL8 (Olympus), or EB‐530US (Fujifilm). After removing the stylet, the specimen was collected by aspiration using a syringe while manipulating the needle back and forth approximately 10 times in the lesion.

### Statistical analysis

The primary endpoint of this study was the overall complication rate, and the secondary endpoints were the overall diagnosis rate of disease, the diagnosis rate of malignant disease, and the treatment rate of malignant disease. The treatment rate was defined as the number of patients who could be treated with tumor‐specific therapy after diagnosis/the number of patients who could be diagnosed by EBUS‐TBNA. Each complication was recorded in the medical record by the treating physician or nurse after the patient had recovered from the procedure. Complications were defined as occurring during the 1‐week follow‐up period and were classified according to the British Thoracic Society guidelines.^9^ The patients requiring treatment as well as requiring hospitalization were included. For hypoxia, the patients with a persistent drop in SpO2 after EBUS‐TBNA were included. In addition, oversedation was defined in this study as a complication of EBUS‐TBNA. Malignant disease was defined as a pathologically diagnosed definitive diagnosis, and benign disease was defined as no significant findings on EBUS‐TBNA and the lesion was not enlarged on computed tomography for more than 6 months. The diagnostic rate was per patient.

## RESULTS

### Patients

**FIGURE 1:**
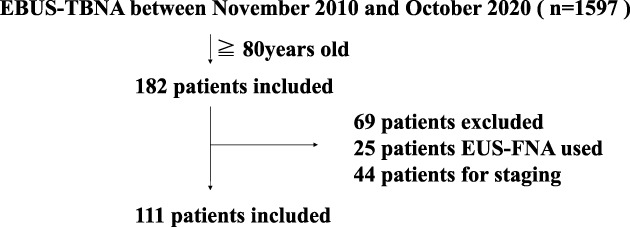
Flow chart of patient enrolment

Of the total number of patients who underwent EBUS‐TBNA during the study period, 180 patients were over 80 years of age. Twenty‐five of the 180 patients underwent endoscopic ultrasound‐guided fine needle aspiration at the same time, and 44 patients underwent staging; therefore, 69 patients were excluded (Figure 1). A total of 111 patients (74 men, 37 women median age: 82 years [range: 80–89 years]) underwent EBUS‐TBNA for diagnostic purposes. The patient characteristics are summarized in Table 1. Eighty‐four patients (76%) had malignant disease, and 27 patients (24%) had benign disease. Most patients had Eastern Cooperative Oncology Group performance status (ECOG PS) score of 0–2, with a small percentage of patients (6%) having ECOG PS score of 3–4.

**TABLE 1:** Patient characteristics

Characteristics	Data (*N* = 111)
Median age, years (range)	82 (80–89)
Gender	
Male (%)	74 (67)
Female (%)	37 (33)
Diagnosis	
Malignant (%)	84 (76)
Benign (%)	27 (24)
Category	
Outpatient (%)	76 (68)
Hospitalization (%)	35 (32)
ECOG PS	
0/1/2/3/4	32/47/25/6/1
ASA PS	
1/2/3/4/5	51/45/14/1/0

Abbreviations: ASA PS, American Society of Anesthesiologists Physical Status; ECOG PS, Eastern Cooperative Oncology Group Performance Status.

### Procedure

Table 2 summarizes the details of the EBUS‐TBNA procedures. The median diameter of the target lesion was 19 mm (range: 8–83 mm), the median number of punctures was 4 (range: 1–9), and the needle size was 22 gauge in 104 cases (94%). Multiple lesions were punctured in 32 cases (29%), and EBUS‐TBNA with rapid on‐site evaluation was performed in 35 cases (32%). The puncture sites are shown in Table 2. The procedure time was available for investigation in 73 cases, with a median time of 20 min 5 s (range 11 min 12 s to 28 min 33 s).

**TABLE 2:** Details of endobronchial ultrasound‐guided transbronchial needle aspiration

Characteristic	Data (*N* = 111)
Median lesion size, mm (range)	19 (8–83)
Median puncture number of main lesion (range)	4 (1–9)
Procedure time	
Sedation	
Midazolam (%)	16 (14)
Midazolam + fentanyl (%)	95 (86)
Needle size	
25G (%)	6 (5)
22G (%)	104 (94)
19G (%)	1 (1)
Bronchoscope	
BF‐UC290F	11 (10)
BF‐UC260‐OL8	93 (84)
EB‐530US	7 (6)
Punctured lesion	
Single (%)	79 (71)
Multiple (%)	32 (29)
ROSE	
With ROSE (%)	35 (32)
Without ROSE (%)	76 (68)
Location of targeted lesion	
#4R/#4 L/#7/#10R/#11R/#11 L	42/10/40/8/9/6
Central parenchyma	25

*Note*: Data are number (range).

Abbreviations: G, gauge; L, left; N, number of patients; R, right; ROSE, rapid on‐site evaluation.

### Safety

The overall number of complications was five, and the primary endpoint, which was the complication rate, was 5%. Complications comprised one case of hypersedation, one case of chest pain, two cases of hypoxia, and one case of arrhythmia. The case of hypersedation was in a patient who received midazolam alone. The five cases that developed complications were summarised as Table 3. The arrhythmia was paroxysmal atrial fibrillation, which required hospitalization for follow‐up. There was no mediastinitis, mediastinal emphysema, pneumothorax, severe bleeding, or procedure‐related death.

**TABLE 3:** Details of patients with complications

	Age Gender	Diagnosis	ECOG PS	Anesthesia	Targeted lesion	Time	Complication
1	82 Male	Malignant lymphoma	1	Midazoram 3 mg	#4R	17 min 8 s	Oversedation
2	81 Female	Lung adenocarcinoma	1	Midazoram 2 mg Fentanyl 50 μg	Central parenchyma	—^a^	Arrhythmia
3	85 Male	Non‐small lung cancer	2	Midazoram 1 mg Fentanyl 50 μg	Central parenchyma	11 min 30 s	Chest pain
4	84 Female	Lung adenocarcinoma	2	Midazoram 1 mg Fentanyl 40 μg	#4R #7	26 min 10 ss	Hypoxia
5	81 Female	Lung metastasis of uterine cancer	0	Midazoram 2 mg Fentanyl 40 μg	Central parenchyma	17 min	Hypoxia

^a^ Patient 2 had no procedure time recorded in the medical record.

### Efficacy

Regarding the secondary endpoints, the diagnostic rate for all patients was 75% (83/111), and the diagnostic rate for malignant disease was 89% (75/84). Sixty‐one of the 75 patients (81%) with malignant disease diagnosed by EBUS‐TBNA received tumor‐specific therapy in accordance with the diagnosis (Table 3). Of the 61 (55%) patients with lung cancer, 58 (95%) were diagnosed by EBUS‐TBNA, and 46 patients (79%) were treated. The lung cancer patients included 22 cases of small cell lung cancer, and the diagnosis rate was 100%. The results for other cancers, such as malignant lymphoma and esophageal cancer, are shown in Table 4. The overall diagnostic rate for malignant diseases was 89%, and 81% of the patients were treated.

**TABLE 4:** Efficacy of endobronchial ultrasound‐guided transbronchial needle aspiration (EBUS‐TBNA)

Diagnosis	Patients (%)	Diagnostic cases (%)	Treated cases (%)
Malignant			
Lung cancer	61 (55)	58 (95)	46 (79)
Malignant lymphoma	12 (11)	7 (58)	7 (100)
Esophageal cancer	13 (3)	2 (67)	2 (100)
Other carcinoma	8 (7)	8 (100)	6 (75)
Benign	27 (24)	8 (30)	
Total	111	83 (75)	

## DISCUSSION

The safety and efficacy of EBUS‐TBNA as a technique for biopsy of lesions close to the trachea or central bronchi, such as mediastinal lymph nodes and mediastinal tumors, have been established.^10^ Favorable results were also obtained specifically in a cohort of elderly patients.^7^ However, no study of EBUS‐TBNA has previously evaluated patients older than 80 years, and to the best of our knowledge, our report is the first in this regard. In our study, 5% of the patients experienced complications after EBUS‐TBNA, which was the primary endpoint of our study. The incidence of complications in our study was similar to that of previous studies in patients younger than 80 years of age,^7^, ^11^, ^12^, ^13^ which confirms the safety of EBUS‐TBNA in patients aged over 80 years (Table 5).

**TABLE 5:** Studies of endobronchial ultrasound‐guided transbronchial needle aspiration (EBUS‐TBNA) in different age groups

Study	Patients	Complications	Diagnostic rate	Age	Design
Demirci et al.^11^	203	3%	97%	65 years or older	Retrospective
Sahajal et al.^12^	248	4%	49%	65 years or older	Retrospective
Okachi et al.^13^	34	3%	96%	70 years or older	Retrospective
Şehnaz et al.^14^	39	8%	46%	70 years or older	Retrospective
Evison et al.^7^	198	5%	86%	70 years or older	Prospective
This study	111	5%	75%	80 years or older	Retrospective

The diagnostic rate of malignant disease, a secondary endpoint of this study, was 89%, and the rate of specific treatment for cancer was 81%. Among lung cancers, the results for small cell lung cancer were particularly good, suggesting that EBUS‐TBNA was indicated for patients over 80 years of age at our institution. Of note, non‐small cell lung cancer is often associated with an improved prognosis in patients with positive driver gene mutations, such as epidermal growth factor receptor (EGFR) mutation and EML4‐ALK gene mutation.^4^, ^15^ As for lung cancer in this study, one patient underwent surgery, two underwent chemoradiation, three underwent radiation therapy, and 40 underwent chemotherapy and other drug therapies. Among them, four patients received molecular targeted drugs and three patients received ICI monotherapy. Among elderly NSCLC patients who opted for best supportive care (BSC), the absence of genetic mutations and low PD‐L1 expression were likely to be the main reasons for choosing BSC. We believe that pathological diagnosis by EBUS‐TBNA may be useful for patients with BSC.

The diagnosis rate of benign diseases was 32%, which was lower than that previously reported.^10^ The low diagnostic rate of benign disease in this study may be due to the high percentage of nonspecific lymph nodes. In addition, this difference may have been caused by selection bias that prioritized imaging follow‐up, such as with computed tomography, over invasive biopsy in the case of benign disease, which is often not prognostic for the elderly, even if EBUS‐TBNA is indicated.

There are several limitations in this study. First, this was a retrospective analysis, which may have led to selection bias. In addition, secondary endpoints indicating efficacy were not assessed, such as the quality of the tissue collected by EBUS‐TBNA, specificity, accuracy, the positive predictive value and negative predictive value. As this was a retrospective study, the bronchoscopists might have evaluated the patients and excluded some cases that were not fit before the procedure, resulting in good ECOG PS patients included in this study.

In conclusion, because the safety of EBUS‐TBNA can be expected in patients over 80 years of age, and these patients may benefit from the test, we believe that there is no need to restrict the age for performing EBUS‐TBNA to those less than 80 years of age.

## CONFLICT OF INTEREST

The authors have stated explicitly that there are no conflicts of interest regarding this article.
